# Telomeric G-quadruplexes are a substrate and site of localization for human telomerase

**DOI:** 10.1038/ncomms8643

**Published:** 2015-07-09

**Authors:** Aaron L. Moye, Karina C. Porter, Scott B. Cohen, Tram Phan, Katherine G. Zyner, Natsuki Sasaki, George O. Lovrecz, Jennifer L. Beck, Tracy M. Bryan

**Affiliations:** 1 Children's Medical Research Institute, University of Sydney, 214 Hawkesbury Road, Westmead, New South Wales 2145, Australia; 2 Commonwealth Scientific and Industrial Research Organisation, Manufacturing Flagship, 343 Royal Parade, Parkville, Victoria 3052, Australia; 3 School of Chemistry, University of Wollongong, Northfields Avenue, Wollongong, New South Wales 2522, Australia

## Abstract

It has been hypothesized that G-quadruplexes can sequester the 3′ end of the telomere and prevent it from being extended by telomerase. Here we purify and characterize stable, conformationally homogenous human telomeric G-quadruplexes, and demonstrate that human telomerase is able to extend parallel, intermolecular conformations *in vitro*. These G-quadruplexes align correctly with the RNA template of telomerase, demonstrating that at least partial G-quadruplex resolution is required. A highly purified preparation of human telomerase retains this extension ability, establishing that the core telomerase enzyme complex is sufficient for partial G-quadruplex resolution and extension. The parallel-specific G-quadruplex ligand *N*-methyl mesoporphyrin IX (NMM) causes an increase in telomeric G-quadruplexes, and we show that telomerase colocalizes with a subset of telomeric G-quadruplexes *in vivo*. The ability of telomerase to partially unwind, extend and localize to these structures implies that parallel telomeric G-quadruplexes may play an important biological role.

## Abstract (web summary)

G-quadruplexes formed by four guanine bases in a square planar arrangement in telomeres may prevent extension of this region by telomerase. Here, the authors show that telomerase can localize to and partially unwind and extend G-quadruplexes, suggesting an important biological role for G-quadruplexes.

## Graphical abstract

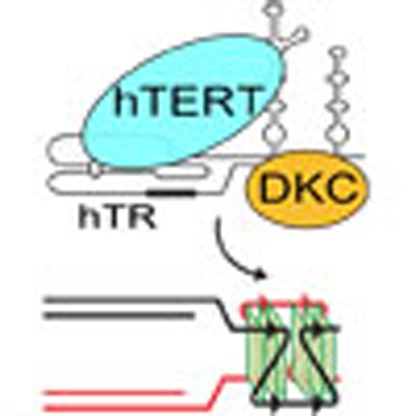

Human chromosomes terminate with ∼5–10 kb of the telomeric DNA repeat TTAGGG^1^,^2^. The shortening of telomeres to a critical length is a signal for cellular senescence^3^,^4^. Telomere shortening is counteracted by telomerase, the telomere-specific reverse transcriptase that contains an RNA template to direct the addition of telomeric DNA by the catalytic telomerase reverse transcriptase (TERT) component^5^,^6^,^7^. Telomerase is upregulated in ≥85% of tumours, providing a telomere maintenance mechanism that contributes to cellular immortalization and tumour progression^8^,^9^.

Telomeric DNA can fold into compact G-quadruplex structures involving the interaction of four guanine bases in a square planar arrangement stabilized by central cations^10^. G-quadruplexes can form from one, two or four strands of DNA in a parallel or antiparallel orientation^11^. Over 375,000 sequences with the potential to form G-quadruplexes have been identified within the human genome^12^. There is increasing evidence supporting *in vivo* functions for DNA and RNA G-quadruplexes, including telomere protection and involvement in transcription, translation and splicing^13^,^14^,^15^,^16^. Direct evidence for the existence of G-quadruplexes at telomeres was obtained using specific antibodies against G-quadruplexes in the telomeric DNA of the ciliate *Stylonychia lemnae*^17^. Recently, a G-quadruplex-specific antibody has also been used to directly visualize G-quadruplexes throughout the human genome, a portion of which is located at telomeres^18^. There are a large number of small-molecule ligands that have specificity for G-quadruplexes, with many of them causing telomere dysfunction *in vivo* and inhibiting telomerase activity *in vitro*^19^,^20^,^21^.

The first report describing the inability of telomerase to extend G-quadruplexes, using the ciliate *Oxytricha nova*, did not differentiate between specific conformations as substrates^22^. In 2006, we demonstrated using purified G-quadruplex structures that some conformations can be extended by telomerase from the ciliated protozoa *Tetrahymena thermophila* and *Euplotes aediculatus*: parallel-stranded intermolecular G-quadruplexes were substrates for ciliate telomerase, whereas antiparallel intramolecular G-quadruplexes were not^23^. Telomere biology is very different between ciliated protozoa and humans, and the telomerase ribonucleoproteins from the two groups differ in key properties such as the ability to extend non-telomeric DNA, the mechanism of template boundary definition and the components and multimeric state of the enzyme complex^24^,^25^,^26^,^27^,^28^. The possibility therefore existed that the ability to extend parallel G-quadruplexes is a ciliate-specific property of telomerase. Given the intense interest in telomerase inhibition by G-quadruplex stabilizers as a potential cancer therapeutic^20^,^21^, and the polymorphism of human telomeric G-quadruplexes^11^, we considered it important to determine the ability of human telomerase to extend defined conformations of G-quadruplex.

In this study, we establish that the ability to extend parallel intermolecular G-quadruplexes is conserved among evolutionarily distant species. We characterize purified, stable, conformationally homogenous human telomeric G-quadruplexes and demonstrate that human telomerase is able to recognize and partially unwind parallel intermolecular conformations and extend the unwound 3′ end *in vitro*. Treatment of immortal human cells with the parallel-specific G-quadruplex ligand *N*-methyl mesoporphyrin IX results in a significant increase in G-quadruplexes present at telomeres *in vivo*, indicating the potential for formation of a parallel G-quadruplex structure at human telomeres. We show that human telomerase localizes to a subset of telomeres that contain G-quadruplexes *in vivo*.

## Results

### Folding and characterization of parallel G-quadruplexes

To test the ability of human telomerase to extend G-quadruplex DNA, we isolated and characterized parallel, intermolecular G-quadruplexes, since this is the conformation extended by ciliate telomerase^23^. To be certain that telomerase extends the G-quadruplex rather than its unfolded linear counterpart, it is necessary to work with structures with very slow unfolding rates under the conditions of a telomerase activity assay (30 °C, 1 h). Human telomeres have one less contiguous guanine residue than their ciliate counterparts (TTAGGG versus TTGGGG), which renders the corresponding G-quadruplexes less stable^29^. It is possible, however, to form stable human telomeric G-quadruplexes using cations such as Sr^2+^, rather than K^+^ or Na^+^ (refs 30, 31). Tetramolecular G-quadruplexes formed from short oligonucleotides also tend to be relatively stable^29^,^32^.

**Figure 1:**
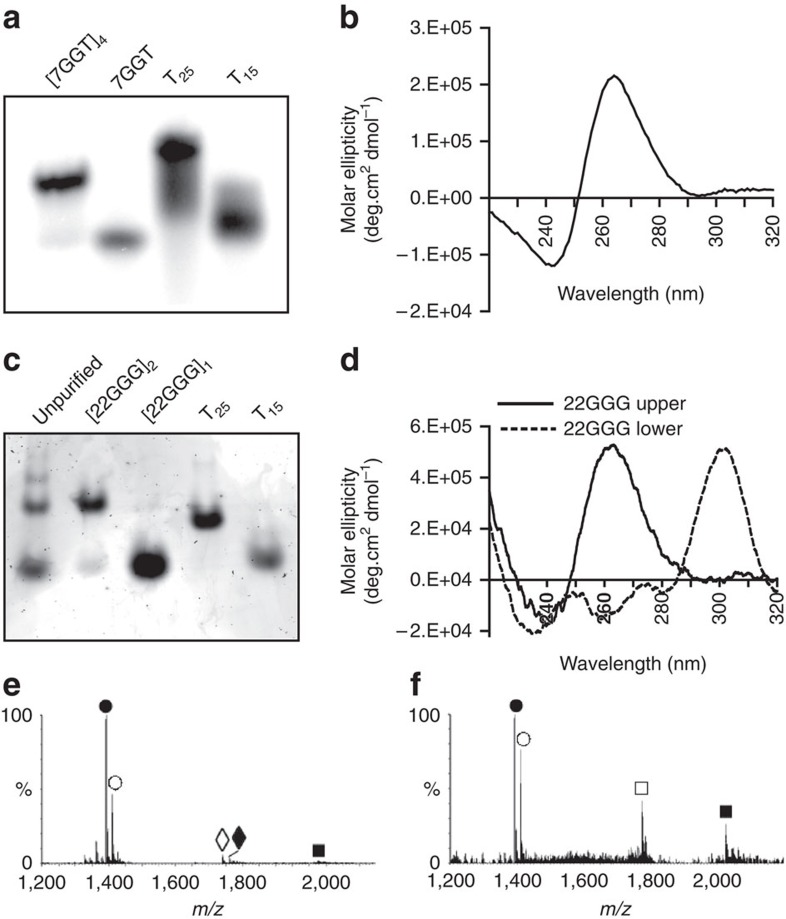
Characterization of gel-purified telomeric G-quadruplexes. (**a**) Native gel electrophoresis of purified ^32^P-labelled [7GGT]_4_ in 150 mM K^+^. Lane 1: [7GGT]_4_ after gel purification. Lane 2: unfolded 7GGT. Lanes 3 and 4: T_25_ and T_15_ unstructured molecular weight (MW) markers. (**b**) CD spectrum of gel-purified [7GGT]_4_ in 150 mM K^+^. (**c**) Native gel electrophoresis of G-quadruplexes formed from 22GGG in 2.5 mM Sr^2+^, stained in SYBR Gold. Lane 1: unpurified folded 22GGG. Lane 2: post-purification folded 22GGG upper band, [22GGG]_2_. Lane 3: post-purification 22GGG lower band, [22GGG]_1_. Lanes 4 and 5: unstructured MW markers T_25_ and T_15_. (**d**) CD spectra of gel-purified 22GGG G-quadruplexes in 2.5 mM Sr^2+^. (**e**) Negative ion ESI mass spectra of the lower band of folded 22GGG and (**f**) the upper band of folded 22GGG. ● [M-5H]^5−^; ◊ [M-4H]^4−^; ○ [M+Sr^2+^-7H]^5−^; ♦ [M+Sr^2+^-6H]^4−^; ▪ [2M+3Sr^2+^-13H]^7−^; □ [2M+3Sr^2+^-14H]^8−^.

The oligonucleotide 7GGT (Table 1) formed a tetrameric G-quadruplex, [7GGT]_4_, when annealed in buffer containing 150 mM KCl, and gave a single band upon native gel electrophoresis. This band was purified from the native gel and remained >98% pure after purification (Fig. 1a). The circular dichroism (CD) spectrum of [7GGT]_4_ gave a positive signal at 260 nm and a negative signal at 240 nm, characteristic of a parallel G-quadruplex (Fig. 1b)^33^. The ultraviolet thermal difference spectrum (TDS)^34^ of [7GGT]_4_ showed a negative peak at 295 nm (Supplementary Fig. 1a), supporting the assignment of a G-quadruplex structure^29^,^32^.

The oligonucleotide 22GGG (Table 1) gave rise to two bands on a native gel when annealed in 2.5 mM Sr^2+^ (Fig. 1c). The migration of the upper and lower bands was consistent with formation of intermolecular and intramolecular structures, respectively^10^. Each G-quadruplex band remained ≥95% pure after gel purification (Fig. 1c); no linear 22GGG was observed after purification. CD analysis of the upper band was indicative of a parallel G-quadruplex structure (Fig. 1d), whereas the lower band gave a maximum absorbance at 295 nm and a minimum at 260 nm, consistent with an antiparallel structure (Fig. 1d). The TDS spectra for both the upper and lower bands of 22GGG confirmed G-quadruplex structures (Supplementary Fig. 1b,c).

Electrospray ionization mass spectrometry (ESI-MS) was used to determine G-quadruplex stoichiometry^35^,^36^. The purified 22GGG lower band gave predominant ions that were assigned to unimolecular species, consistent with the inclusion of an Sr^2+^ ion within an intramolecular G-quadruplex (Fig. 1e; Supplementary Table 1). A lower-abundance ion can be assigned as the dimeric G-quadruplex species [2M+3Sr^2+^-13H]^7−^ (where M is neutral, unimolecular 22GGG), consistent with the observation of some reformation of the upper parallel G-quadruplex after purification. Together, the migration pattern on native gel electrophoresis, ESI-MS and CD analysis indicate that the lower band of 22GGG annealed in Sr^2+^ is an intramolecular antiparallel G-quadruplex, which will henceforth be referred to as [22GGG]_1_.

The predominant ions observed from the purified upper band of 22GGG represented bimolecular DNA along with a unimolecular G-quadruplex (Fig. 1f; Supplementary Table 1); this is consistent with the observation of some upper band converting to the intramolecular G-quadruplex after purification. The ESI mass spectrum, combined with CD and TDS analysis and native gel electrophoresis, is consistent with a parallel, dimeric G-quadruplex, here called [22GGG]_2_.

### Absence of spontaneous unfolding of G-quadruplexes

The relative stabilities of the purified G-quadruplexes were initially assessed by determining their melting temperatures. Since intermolecular G-quadruplexes reform very slowly and are therefore not at equilibrium during the measurement, we refer to this value as *T*_½_ rather than *T*_m_, which is the true thermodynamic parameter at equilibrium^29^. The ultraviolet melting curve of [7GGT]_4_ gave a *T*_½_ of 68 °C (measured at a heating rate of 1 °C min^−1^; Supplementary Fig. 1d; Table 1).

**Figure 2:**
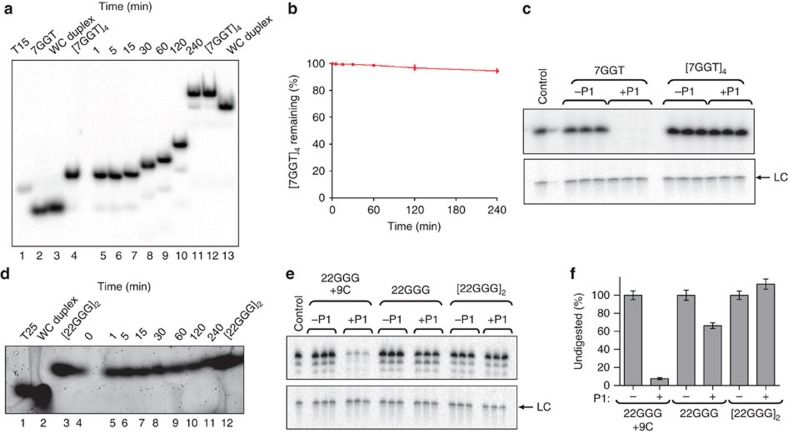
Stability of telomeric G-quadruplexes. (**a**) [7GGT]_4_ complementary strand trap assay: 5′-end-labelled [7GGT]_4_ in 150 mM K^+^, incubated with 10-fold excess complementary strand 9C at 30 °C and electrophoresed on a 12% non-denaturing polyacrylamide gel. Lane 1: unstructured molecular weight (MW) marker T_15._ Lane 2: denatured 7GGT. Lane 3: pre-annealed Watson–Crick (WC) duplex. Lane 4: gel-purified [7GGT]_4_ G-quadruplex. Lanes 5–11: [7GGT]_4_ incubated with 9C at 30 °C for the indicated times. Lane 12: [7GGT]_4_. Lane 13: pre-annealed WC duplex. Samples were loaded at the times indicated, with the gel running between loadings, so later samples underwent shorter electrophoresis times. The last three lanes were loaded simultaneously. (**b**) Proportion of [7GGT]_4_ remaining over time at 30 °C. Error bars represent s.d. of the mean of three independent experiments. (**c**) P1 digestion of 7GGT and [7GGT]_4_ electrophoresed on a 12% denaturing polyacrylamide gel; digestions were performed in triplicate. Control lane: end-labelled 7GGT. (**d**) [22GGG]_2_ complementary strand trap assay. Unlabelled [22GGG]_2_ in 2.5 mM Sr^2+^, incubated with 10-fold excess complementary strand 9C at 30 °C, electrophoresed on a 16% non-denaturing polyacrylamide gel and stained in SYBR Gold. Lane 1: unstructured MW marker T_25._ Lane 2: pre-annealed WC duplex. Lane 3: gel-purified [22GGG]_2._ Lanes 4–11: G-quadruplex [22GGG]_2_ incubated with 9C at 30 °C for the indicated times. Lane 12: gel-purified [22GGG]_2_. (**e**) P1 digestion of 22GGG hybridized to complementary strand 9C, linear 22GGG and [22GGG]_2,_ electrophoresed on a 12% denaturing polyacrylamide gel; digestions were performed in triplicate. Control lane: end-labelled 22GGG. (**f**) Data from **e**, quantified. Error bars represent s.d. of the mean of triplicates. LC indicates a ^32^P-labelled 100-nt oligonucleotide used as a control for loading and recovery.

To determine the amount of [7GGT]_4_ that unfolds during the course of a telomerase activity assay (30 °C, 1 h), we employed a complementary C-strand trap assay. A 10-fold excess of C-rich complementary strand (Table 1) was added to the G-quadruplex in the same buffer used for telomerase activity assays (described below), and the extent of Watson–Crick duplex formation was monitored over 4 h by non-denaturing gel electrophoresis^37^,^38^ (Fig. 2a). Control experiments established that the result was unaffected by increasing C-strand concentrations, up to 100-fold over G-quadruplex (Supplementary Fig. 2a), demonstrating that the rate of Watson–Crick duplex formation reflects the rate of G-quadruplex unfolding. After 1 h at 30 °C, ∼1% of the G-quadruplex had hybridized to the complementary strand, with ∼3–4% hybridized after 4 h (Fig. 2a,b). As a further test of the stability of [7GGT]_4_, the G-quadruplex was treated with phosphodiesterase 1 (P1), which cleaves single-stranded DNA exonucleolytically at the 3′ end. Under conditions identical to the telomerase activity assays below (30 °C for 1 h), all of the P1-treated linear 7GGT was digested while no [7GGT]_4_ digestion was detected (Fig. 2c). The 7-nt oligonucleotide remained intact after digestion, demonstrating that even the single thymidine at the 3′ end of each of the four strands is participating in tertiary structure and being protected from digestion. Overall, these data demonstrate that the G-quadruplex [7GGT]_4_ is stable under the conditions of the telomerase activity assay.

The *T*_½_ values of [22GGG]_2_ and [2GGG]_1_ were >90 and 68 °C, respectively, when measured at 1 °C min^−1^ (Supplementary Fig. 1e,f; Table 1). The high melting temperature of the dimeric species indicates that it is an exceptionally stable structure. An identical complementary C-strand experiment was performed with [22GGG]_2_ in 2.5 mM Sr^2+^ buffer. After 4 h at 30 °C, there was no observable duplex formation, indicating that the [22GGG]_2_ G-quadruplex does not unfold under our assay conditions (Fig. 2d). This was confirmed by digestion with P1 at 30 °C for 1 h; no digestion of [22GGG]_2_ was observed (Fig. 2e,f, right lanes). It should be noted that any [22GGG]_2_ that did unfold over days or weeks in storage rapidly formed [22GGG]_1_, with no linear 22GGG oligonucleotide observed (Fig. 1c).

Intramolecular G-quadruplexes often display rapid folding and unfolding rates^23^,^39^. Under the conditions used in this study, it was not possible to obtain a native gel or CD spectrum that corresponded to linear, unfolded 22GGG in the presence of 2.5 mM SrCl_2_. When incubated with P1, only ∼30% of ‘linear' 22GGG in 2.5 mM SrCl_2_ was digested (Fig. 2e,f, middle lanes). This suggests that linear 22GGG in the presence of Sr^2+^ is in rapid equilibrium with [22GGG]_1_. To confirm this, we ‘locked' linear 22GGG into an unfolded conformation by pre-binding it with the 9C complementary oligonucleotide (Supplementary Fig. 2b), as previously described^39^. After digestion with P1, only 10% of the DNA remained undigested (Fig. 2e,f, left lanes). These data demonstrate that the P1 exonuclease was functional in this experiment, and that linear 22GGG and intramolecular [22GGG]_1_ G-quadruplex are in rapid equilibrium when in the presence of 2.5 mM Sr^2+^.

### Parallel G-quadruplexes are substrates of human telomerase

**Figure 3:**
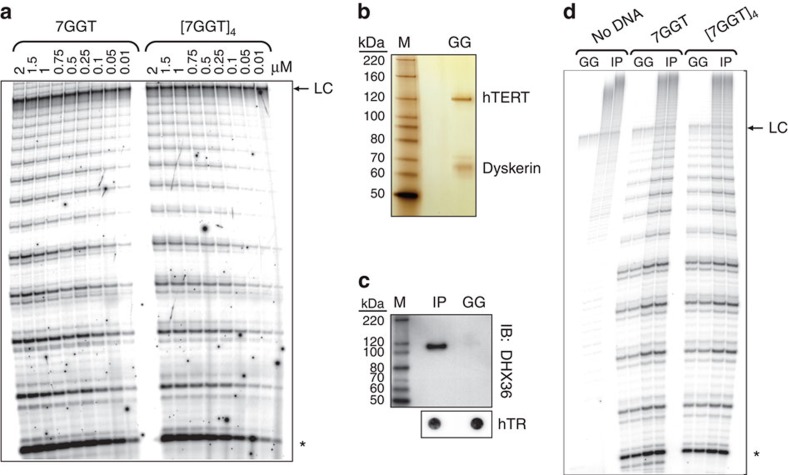
Extension of 7GGT and [7GGT]_4_ by human telomerase. (**a**) Telomerase activity assays with immunopurified telomerase and the indicated concentrations of either 7GGT or [7GGT]_4_. The concentrations of [7GGT]_4_ refer to concentrations of the assembled, four-stranded G-quadruplex. (**b**) Silver-stained SDS–PAGE gel of highly purified telomerase (GG). (**c**) Immunoblot for DHX36 with immunopurified (IP) or highly purified (GG) telomerase. The bottom panel shows a dot-blot northern for hTR, demonstrating equal loading of telomerase in the gel. (**d**) Telomerase activity assays with 2 μM 7GGT or [7GGT]_4_ with immunopurified (IP) or highly purified (GG) telomerase, using equal amounts of each enzyme; reactions were performed in duplicate. For **a** and **d**, the asterisk indicates the first visible addition product (*n*+3); LC indicates a ^32^P-labelled 100-nt oligonucleotide used as a control for loading and recovery.

Telomerase activity assays were performed with the G-quadruplexes characterized above to determine whether parallel, intermolecular G-quadruplexes are a substrate for human telomerase. Telomerase was prepared by overexpressing its components in 293T cells^40^ and immunopurifying the assembled complex with an hTERT antibody^41^. The G-quadruplex [7GGT]_4_ was readily extended by human telomerase (Fig. 3a): the Michaelis–Menton constant (*K*_m_) for 7GGT was 100±20 nM and that for [7GGT]_4_ was 500±120 nM (mean±s.d.; *n*=4–6). Notably, both substrates supported equal amounts of catalytic activity, with a relative *V*_max_ (folded/linear) of 0.97±0.07 (mean±s.d.; *P*=0.3, one-sample *t*-test; *n*=7).

There is a possibility that the one-step telomerase immunopurification method used in these experiments could allow co-immunopurification of other proteins that may selectively unwind the parallel G-quadruplexes, allowing the observed extension. To exclude this possibility, we purified human telomerase further with a four-step procedure involving immunopurification, capture onto an immobilized telomeric DNA substrate, release of active enzyme and sedimentation over a glycerol gradient^42^ (Fig. 3b); the specific activity of telomerase on an 18-nt telomeric substrate does not change over the course of purification (Supplementary Fig. 3). The purity of this telomerase was confirmed on a silver-stained SDS–polyacrylamide gel electrophoresis (PAGE) gel (Fig. 3b); the only visible protein bands correspond to the sizes of the core telomerase components hTERT and dyskerin^42^. The G-quadruplex helicase DHX36 (also known as G4R1 or RHAU) has been shown to associate with a portion of active telomerase in immortal human cells^43^,^44^; we confirmed this association in our 293T system by immunoblot analysis for DHX36 on one-step immunopurified telomerase (Fig. 3c). However, our four-step purification procedure resulted in loss of DHX36, confirmed by immunoblot of an equal molar amount of highly purified telomerase (GG in Fig. 3c).

Telomerase activity assays were performed with this highly purified telomerase alongside one-step immunopurified telomerase. The relative activity using [7GGT]_4_ G-quadruplex and 7GGT linear DNA as substrates was the same for highly purified as for one-step purified telomerase (Fig. 3d). This confirms that the core telomerase enzyme complex is sufficient for extension of [7GGT]_4_.

**Figure 4:**
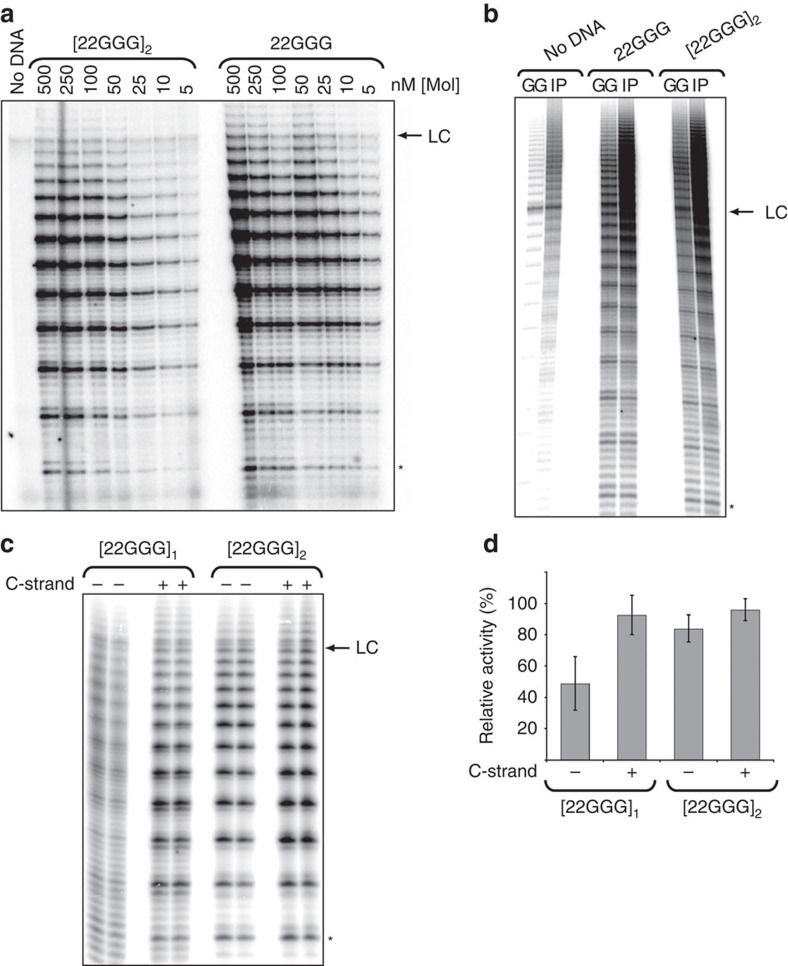
Extension of linear 22GGG and [22GGG]_2_, but not [22GGG]_1_, by human telomerase. (**a**) Telomerase activity assays with immunopurified telomerase and the indicated concentrations of either linear 22GGG or [22GGG]_2_. (**b**) Telomerase activity assays with 1 μM linear 22GGG or [22GGG]_2_ with immunopurified (IP) or highly purified (GG) telomerase, using equal amounts of each enzyme. (**c**) Telomerase activity assays with immunopurified telomerase and 1 μM [22GGG]_1_ and [22GGG]_2_, with or without complementary strand 9C as indicated; reactions were performed in duplicate. (**d**) Quantification of **c** normalized against 22GGG pre-annealed with 9C. Error bars represent s.d. of the mean of three independent experiments. In all panels, the asterisk indicates the first visible addition product (*n*+4), and LC indicates a ^32^P-labelled 100-nt oligonucleotide used as a control for loading and recovery.

The dimeric parallel G-quadruplex [22GGG]_2_ was also readily extended by immunopurified human telomerase, with a *K*_m_ of 53±10 nM (Fig. 4a; mean±s.d.; *n*=4), under conditions in which we had demonstrated that this G-quadruplex is extremely stable. [22GGG]_2_ was also extended by the highly purified telomerase preparation (Fig. 4b). Thus, the core human telomerase complex is capable of extending multiple parallel, intermolecular G-quadruplexes.

Obtaining reproducibly quantitative telomerase activity assays for linear 22GGG and [22GGG]_1_ was confounded by the rapid interconversion between the folded and unfolded forms. It is well established that intramolecular, antiparallel G-quadruplexes are not good substrates for either ciliate or human telomerase^22^,^23^,^39^. To confirm that this is also the case for the [22GGG]_1_ intramolecular G-quadruplex folded in Sr^2+^, we again ‘locked' linear 22GGG in Sr^2+^ into the unfolded form by hybridizing it with the complementary DNA oligonucleotide 9C^39^, which resulted in a twofold recovery of activity (Fig. 4c,d). In contrast, addition of the complementary strand to the [22GGG]_2_ G-quadruplex did not affect activity (Fig. 4c,d). These data support our hypothesis that unfolded 22GGG is in equilibrium between linear and antiparallel G-quadruplex conformations, and confirm that the antiparallel [22GGG]_1_ G-quadruplex is a less-favourable substrate for human telomerase than its linear counterpart. Taken together, these data demonstrate that multiple parallel, intermolecular G-quadruplexes are substrates for human telomerase, while an antiparallel, intramolecular G-quadruplex is not, despite the much higher stability of the former G-quadruplexes. We have also demonstrated that the core human telomerase enzyme complex is sufficient for parallel G-quadruplex extension.

### Telomerase partially unwinds parallel G-quadruplexes

**Figure 5:**
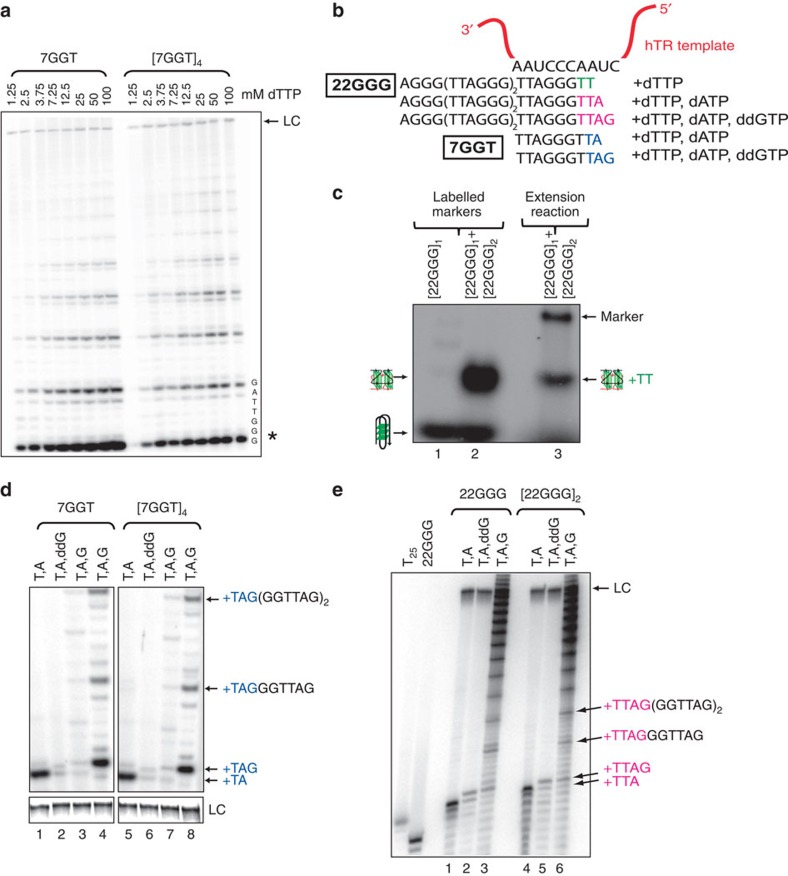
Telomerase partially unwinds and extends [7GGT]_4_ and [22GGG]_2_. (**a**) Telomerase activity assays with immunopurified telomerase, using 1 μM 7GGT or [7GGT]_4_ and the indicated concentrations of dTTP. The asterisk indicates the first visible addition product (*n*+3). (**b**) Expected telomerase products upon alignment of 7GGT or 22GGG with the RNA template in the presence of different combinations of nucleotides. (**c**) Telomerase activity assay followed by native gel electrophoresis. Lane 1: 5′-end-labelled [22GGG]_1_. Lane 2: mixture of 5′-end-labelled [22GGG]_1_ and [22GGG]_2_, demonstrating separation of the two species. Lane 3: products of unlabelled [22GGG]_1_ and [22GGG]_2_ following telomerase extension in the presence of [α-^32^P]-dTTP. Unstructured marker added for orientation purposes. (**d**) Templated addition of nucleotides to 7GGT and [7GGT]_4_. G-quadruplexes and their linear counterparts (2 μM) were incubated with telomerase in the presence of: lanes 1 and 5: ^32^P-dTTP and dATP; lanes 2 and 6: ^32^P-dTTP, dATP and ddGTP; lanes 3 and 7: ^32^P-dTTP, dATP and dGTP; lanes 4 and 8: dTTP, dATP and ^32^P-dGTP, and products electrophoresed on a denaturing 12% acrylamide gel. Migration position of different products is shown. (**e**) Templated addition of nucleotides to 22GGG and [22GGG]_2_. G-quadruplexes and their linear counterparts (2 μM) were incubated with telomerase in the presence of: lanes 1 and 4: ^32^P-dTTP and ddATP; lanes 2 and 5: ^32^P-dTTP, dATP and ddGTP; lanes 3 and 6: dTTP, dATP and ^32^P-dGTP, and products electrophoresed on a denaturing 12% acrylamide gel. End-labelled T_25_ and 22GGG markers are on the left of the gel. Migration position of different products is shown. In all panels, LC indicates a ^32^P-labelled 100-nt oligonucleotide used as a control for loading and recovery.

We have demonstrated that, for *Tetrahymena* telomerase, parallel G-quadruplex substrates increase the *K*_m_ of telomerase for the incoming dTTP nucleotide, presumably by perturbation of the nucleotide-binding site by the wider G-quadruplex substrate^37^. The *K*_m_ of human telomerase for dTTP was determined in the presence of saturating [7GGT]_4_ G-quadruplex and 7GGT linear DNA at a range of dTTP concentrations (Fig. 5a). The *K*_m_ of telomerase for dTTP increased approximately fivefold in the presence of the G-quadruplex substrate, from 2.8±0.5 μM for linear 7GGT to 15±5 μM with [7GGT]_4_ (mean±s.d.; *P*=0.0136, unpaired two-tailed *t*-test; *n*=3). This change in *K*_m_ provides evidence that the observed extension is occurring with two conformationally distinct substrates.

We demonstrated the maintenance of G-quadruplex structure following telomerase extension directly, by visualizing telomerase extension products by native gel electrophoresis. A mixture of unlabelled [22GGG]_1_ and [22GGG]_2_ was extended by telomerase in the presence of [α-^32^P]-dTTP (Fig. 5b,c). Under native gel electrophoresis conditions, a single band was present following telomerase extension (Fig. 5c, lane 3) that aligns with an authentic ^32^P-labelled [22GGG]_2_ marker (Fig. 5c, lane 2). These data provide evidence that the [22GGG]_2_ parallel G-quadruplex structure is maintained following extension by telomerase. Direct observation of [22GGG]_2_ specifically labelled through extension by telomerase provides unequivocal evidence that G-quadruplex DNA must be the substrate utilized by telomerase.

The telomerase RNA template hybridizes to telomeric DNA to enable telomere extension with the correct alignment. Ciliate telomerase can perform extension of non-telomeric substrates without any complementarity between the DNA primer and RNA template, using a specific template position as the default for the initiation of extension^26^. We wished to test whether the extension of parallel G-quadruplexes is preceded by canonical template hybridization (Fig. 5b). We performed telomerase activity assays in the presence of different combinations of nucleotides, for both [7GGT]_4_ and [22GGG]_2_ (Fig. 5d,e). A standard processive reaction including [α-^32^P]-dGTP, dATP and dTTP provided a marker for band positions, as the major pause site in a processive reaction is at the end of the template (products ending in –TAG; see labelling of gel in Fig. 5d,e). In the presence of only [α-^32^P]-dTTP and dATP, we anticipated the addition of two nucleotides (TA) and three nucleotides (TTA) to primers 7GGT and 22GGG, respectively, if the terminal 3′ nucleotides are correctly aligning with the RNA template (Fig. 5b). Because addition of ddGTP results in termination of DNA strand elongation, we anticipated the addition of three nucleotides (TAG) and four nucleotides (TTAG) to 7GGT and 22GGG, respectively, in the presence of [α-^32^P]-dTTP, dATP and ddGTP. In all cases, the patterns of extension of parallel G-quadruplexes [7GGT]_4_ and [22GGG]_2_ are identical to their respective linear controls (Fig. 5d,e) and correspond with expected nucleotide addition patterns (Fig. 5b). For canonical hybridization to occur, at least a portion of the parallel G-quadruplex structure must be unwound. As native gel electrophoresis demonstrated alignment of the extended parallel G-quadruplex with an identical ^32^P-labelled marker (Fig. 5c), we conclude that parallel G-quadruplexes are partially unwound to allow hybridization of the telomerase template with the 3′ end of the DNA.

### Telomerase localizes to G-quadruplex-containing telomeres

**Figure 6:**
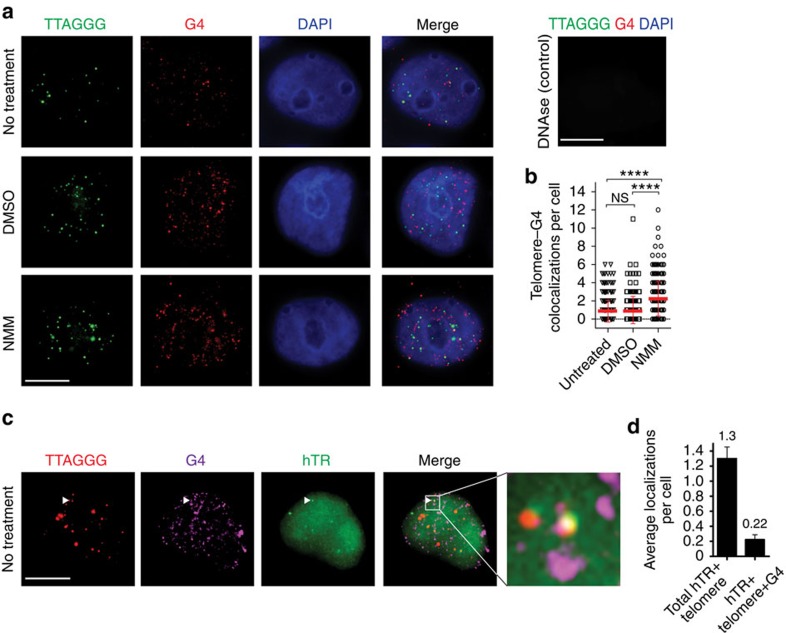
G-quadruplex-containing telomeres are a site of localization for human telomerase. (**a**) Immunofluorescence with G-quadruplex antibody BG4 together with telomere FISH, in mid-S-phase HEK 293T cells with no treatment (top), vehicle (dimethyl sulfoxide (DMSO), middle) or parallel-specific ligand *N*-methyl mesoporphyrin IX (NMM) (bottom). DNAse treatment is included as a G-quadruplex antibody control (top right). (**b**) Quantification of number of telomere-G-quadruplex colocalizations per cell from **a**. One hundred nuclei were counted per condition in two (DMSO) or three (untreated and NMM) independent experiments. Error bars represent the s.e.m. Statistical significance was calculated using an unpaired *t*-test; ****P<0.0001 (**c**) Immunofluorescence with G-quadruplex antibody BG4 together with FISH for telomeres and telomerase RNA (hTR). (**d**) Quantification of hTR–telomere–BG4 trilocalization frequency, relative to telomerase–telomere localization events. Hundred nuclei were counted in each of the three independent experiments. Error bars represent the s.e.m. Scale bars, 10 μm. NS, not significant.

To investigate the presence of parallel telomeric G-quadruplexes in cells, we performed immunofluorescence on human embryonic kidney 293T cells using a G-quadruplex-specific antibody^18^. Fixed cells were subjected to cytoplasmic extraction prior to immunofluorescence to remove RNA G-quadruplexes; a DNase-treated control demonstrates the specificity of the antibody for DNA G-quadruplexes under these conditions (Fig. 6a, top right). A subset of telomeres colocalized with G-quadruplex foci (Fig. 6a, top panel), as previously demonstrated^18^. Treatment of the cells with the parallel-specific G-quadruplex porphyrin ligand *N*-methyl mesoporphyrin IX^45^,^46^,^47^ (Supplementary Fig. 4a) during mid-S phase of the cell cycle resulted in an increase in the number of G-quadruplex–telomere colocalizations per cell (Fig. 6a,b), without affecting progression of the cells into mid-S phase (Supplementary Fig. 4b). These data provide evidence that parallel G-quadruplexes can form at human telomeres *in vivo*.

Since human telomerase can extend parallel G-quadruplexes *in vitro*, we asked whether telomeric G-quadruplexes are a site of localization for human telomerase *in vivo*. 293T cells were synchronized in mid-S phase, which is the peak of telomerase recruitment to telomeres^48^. Using G-quadruplex immunofluorescence, combined with fluorescence *in situ* hybridization (FISH) for telomerase RNA (hTR) and telomeres^49^, we observed the simultaneous colocalization of endogenous telomerase, G-quadruplexes and telomeres (Fig. 6c; Supplementary Fig. 5). Control experiments demonstrate that depletion of dyskerin, a component of the core telomerase complex^42^, markedly reduces hTR foci, verifying the specificity of the FISH signal (Supplementary Fig. 6). G-quadruplex foci colocalized with ∼17% of total hTR-telomere foci (Fig. 6d). These results reveal that human telomerase can localize to telomeres at which G-quadruplexes are present.

## Discussion

It has been hypothesized that G-quadruplexes can sequester the 3′ end of the telomere and prevent it from being extended by telomerase^22^,^39^. Here, we demonstrate that parallel intermolecular telomeric G-quadruplexes are partially unwound and robustly extended by human telomerase. We used highly purified telomerase to show that the core telomerase enzyme complex has the ability to partially unwind the G-quadruplexes prior to their extension.

**Figure 7:**
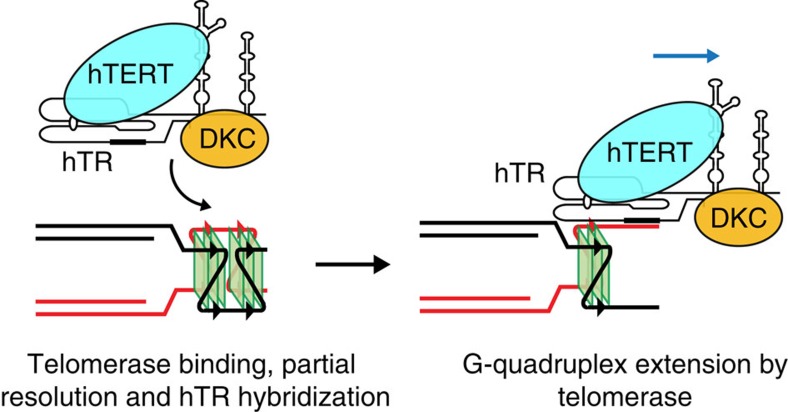
Proposed model for telomerase action at telomeric G-quadruplexes. Telomerase binds to a parallel G-quadruplex structure. The core telomerase complex is then able to partially unwind the G-quadruplex allowing hybridization of the RNA template and DNA extension.

The apparent affinity of telomerase for the incoming dTTP nucleotide was significantly reduced when extending [7GGT]_4_ compared with linear 7GGT, suggesting a change in the conformation of (or near) the active site of telomerase to accommodate the wider G-quadruplex structure. This difference of *K*_m_ with different conformations of DNA substrate demonstrates that the parallel G-quadruplex is a substrate for telomerase while still at least partially structured, an observation we confirmed using native gel electrophoresis of a parallel G-quadruplex following extension (Fig. 5c). Nevertheless, the telomerase extension patterns demonstrate correct alignment of the 3′ end of the DNA substrate with the RNA template, indicating that parallel G-quadruplexes are partially unwound by or invaded by telomerase. Neither of the parallel G-quadruplexes in this study demonstrated any hybridization to a complementary DNA oligonucleotide or digestion by a nuclease under the conditions of the telomerase activity assays, arguing against a model of spontaneous transient unfolding of the DNA 3′ ends followed by telomerase extension. Given the exceptional stability of the [22GGG]_2_ G-quadruplex, in particular, these data support an extension model where the parallel G-quadruplex structure is bound and subsequently partially resolved by telomerase prior to extension of the DNA 3′ end (Fig. 7). We are currently investigating the mechanism of this resolution, including the regions of telomerase responsible, given that neither hTERT nor dyskerin contains a known helicase domain. It is possible that telomerase uses a similar mechanism as that proposed for replication protein A, involving binding to the single-stranded loop regions between stacks of G-quartets^50^.

Currently, there are a large number of laboratories investigating the interactions of stabilizing ligands with G-quadruplexes. It is therefore crucial to determine the specificity of human telomerase for the different conformations of G-quadruplexes, such that once the *in vivo* structure(s) of human telomeres is determined, suitable G-quadruplex-stabilizing ligands of appropriate specificity can be rationally selected. Although *in vivo* evidence for G-quadruplex structures is increasing, the structure-specific locations and functions of G-quadruplexes remain speculative. Our *in vivo* data indicate that parallel G-quadruplexes can form at human telomeres, and that telomeres containing a G-quadruplex are a site of localization for human telomerase. That telomerase is also able to partially unwind and extend these structures, and the evolutionary conservation of this property in distantly related organisms such as *Tetrahymena*^23^, implies that telomeric DNA in a parallel G-quadruplex conformation may have a biological role. In support of this idea, it has been demonstrated that promotion of parallel telomeric G-quadruplexes by the *Saccharomyces cerevisiae* protein Est1p is essential for telomerase-mediated telomere elongation in that organism^51^. One potential biological role for telomeric G-quadruplexes in human cells may be during meiosis, when two pairs of sister chromatids are brought together; it has been suggested that parallel G-quadruplexes could be responsible for the correct alignment of the four chromatids^52^. The ability of telomerase to partially unwind and extend parallel G-quadruplexes potentially formed during meiosis may be necessary for the maintenance of germline telomere length. Alternatively, G-quadruplexes may be involved in the association of telomeres of sister chromatids during S phase, which is known to be necessary for telomerase to lengthen telomeres in human cells^53^. The data presented here provide evidence that subtypes of telomeric G-quadruplexes interact differentially with human telomerase, possibly reflecting their different biological roles.

## Methods

### Oligonucleotide preparation

DNA oligonucleotides (Table 1) were purchased from Sigma Genosys in desalted form. All oligonucleotides were purified by electrophoresis on denaturing 20% polyacrylamide/8 M urea gels in 1 × TBE buffer (89 mM Tris, 89 mM borate and 2 mM EDTA). The major band was excised and eluted by crushing and soaking for 12–16 h at 4 °C with rotation in TEK (10 mM Tris-HCl, pH 7.5–8.0, 1 mM EDTA and 250 mM KCl) for 7GGT and TESr (10 mM Tris-HCl, pH 7.5–8.0, 1 mM EDTA and 250 mM SrCl_2_) for 22GGG, and ethanol precipitated for 2–16 h at −20 °C. The precipitated product was resuspended in 10 mM Tris-Cl, pH 7.5.

### G-quadruplex formation and purification

7GGT (1 mM) or 22GGG (700 μM) were heat denatured in K^+^ hTel buffer (50 mM Tris-HCl, pH 8.0, 1 mM MgCl_2_ and 150 mM KCl) or Sr^2+^ hTel buffer (50 mM Tris-HCl, pH 8.0, 1 mM MgCl_2_ and 2.5 mM SrCl_2_), respectively, for 5 min at 95 °C. They were allowed to cool slowly (∼1 h) to 25 °C and left to equilibrate at this temperature for 72 h. The folded DNA was added to 6 × native gel loading buffer (0.25% bromophenol blue, 0.25% xylene cyanol and 30% glycerol). 7GGT was electrophoresed on a non-denaturing 12% polyacrylamide gel containing 150 mM KCl for 4.5 h at 12 W at 22 °C. 22GGG was electrophoresed on a non-denaturing 12% polyacrylamide gel containing 2.5 mM SrCl_2_ for 24 h at 40 V at 18 °C. Both buffer and gel contained the same constituents as the DNA-folding buffer. Ultraviolet shadowing was used to confirm the location of the DNA bands on the gel. The band of interest was excised and crushed in either TEK (7GGT) or TESr (22GGG) and incubated for 2–16 h at 4 °C with rotation. The supernatant was filtered (0.22 μm) and the DNA precipitated with ethanol for 2–16 h at −20 °C. The precipitated product was resuspended in the original folding buffer. DNA concentrations were determined by ultraviolet absorbance at 260 nm (7GGT: 69,800 M^−1^ cm^−1^; 22GGG: 228,500 M^−1^ cm^−1^; 9C: 76,500 M^−1^ cm^−1^). Concentrations of G-quadruplexes are given as the concentration of assembled complexes (that is, taking strand stoichiometry into account). Folded G-quadruplexes were stored at 4 °C until use.

In some experiments, oligonucleotides were 5′-end-labelled with [γ-^32^P]ATP prior to G-quadruplex formation and purification, as described^23^. Radiolabelled structures were used for some native gel analyses (for example, Fig. 1a), and P1 digestion assays, at 10–15 × 10^3^ c.p.m. per gel lane, whereas unlabelled G-quadruplexes were used for telomerase assays, CD analysis and other native gel analyses (for example, Fig. 1c). In the latter experiment, 250 ng of each DNA was electrophoresed on a non-denaturing 12% polyacrylamide gel, which was stained in 1 × SYBR Gold (Life Technologies) at 25 °C for 30 min, and visualized on a Typhoon FLA9500 scanner (GE Healthcare Lifesciences) using a 488-nm laser and a 526 BP emission filter.

### Circular dichroism

CD spectra were recorded at 25 °C on a Jasco J-810 spectrometer or an Aviv 215S CD spectrometer equipped with a Peltier temperature controller. G-quadruplex samples of the desired conformation were prepared at 20 μM in corresponding hTel buffers. Four scans were accumulated over the wavelength range 220–320 nm in a 0.1-cm pathlength cell at standard sensitivity, data pitch 0.1 nm, continuous scanning mode, scanning speed 100 nm min^−1^, response 4 s and bandwidth 1 nm. Buffers alone were also scanned and these spectra subtracted from the average scans for each sample. CD spectra were collected in units of millidegrees, normalized to the total species concentrations and expressed as molar ellipticity units (deg × cm^2^ dmol^−1^).

### Electrospray ionization mass spectrometry

All ESI mass spectra were obtained using a Waters Q-TOF Ultima ESI mass spectrometer (Manchester, UK). Purified G-quadruplex samples were dissolved in 150 mM NH_4_OAc at a concentration of 20 μM. In all experiments the capillary voltage (2.2 kV), cone voltage (35 V), desolvation temperature (40 °C), radio frequency lens (65 V), desolvation gas flow (150 l h^−1^), collision energy (4 V), cone gas flow (0.0021, l h^−1^) and TOF (9.1 kV) remained the same. All spectra were obtained in the negative ion mode. The instrument was calibrated using 1 mg ml^−1^ caesium iodide. Samples were injected at a flow rate of 10 μl min^−1^ with a Harvard Model 22 syringe pump (Natick, USA).

### Complementary C-strand trap assay

This method was carried out using a modified procedure to that published^37^,^38^. ^32^P-labelled gel-purified [7GGT]_4_ (3,000 c.p.m. μl^−1^, 4 μM) or unlabelled [22GGG]_2_ (2.5 μM) were incubated in the presence of a 10-fold excess of 9C (Table 1) at 30 °C to give a final reaction volume of 28 μl. Aliquots (4 μl) of this hybridization reaction were removed at regular time intervals and loaded onto a native 12% polyacrylamide gel containing 150 mM KCl ([7GGT]_4_) or a native 16% polyacrylamide gel containing 2.5 mM SrCl_2_ ([22GGG]_2_), with the gel running continuously between time points. Electrophoresis was conducted as described above. For complementary C-strand trap assays with increasing 9C concentrations, ^32^P-labelled gel-purified [7GGT]_4_ was incubated at 11 μM in the presence of a 5-, 10-, 25- or 100-fold excess of 9C at either 30 °C or 37 °C. For [7GGT]_4_, the gel was dried at 80 °C for 40 min, exposed to a PhosphorImager screen, visualized on a Typhoon FLA9500 scanner and analysed using ImageQuant software. For [22GGG]_2_, the gel was stained with SYBR Gold at 25 °C for 30 min, visualized on a Typhoon FLA9500 scanner and analysed using ImageQuant software. Uncropped versions of blots are provided in Supplementary Fig. 7.

### Phosphodiesterase 1 digestion of G-quadruplexes

^32^P-end-labelled [7GGT]_4_ or [22GGG]_2_ (15,000 c.p.m.) and their linear controls were incubated with 2 μg P1 (Affymetrix; resuspended in 110 mM Tris-HCl, pH 8.9, 110 mM NaCl, 15 mM MgCl_2_ and 50% glycerol) in hTel buffer (as listed above) for 1 h at 30 °C in a total reaction volume of 20 μl. The reaction was terminated with 80 μl of stop-buffer (50 mM Tris-HCl, pH 8.3, 20 mM EDTA, 0.2% SDS and 1–2 × 10^3^ c.p.m. of a 5′-^32^P-labelled synthetic 100-mer DNA as an internal recovery standard). From this point, the samples were treated in the same manner as ‘telomerase activity assay' reactions (see below).

### HEK293T fermentation and telomerase overexpression

HEK293T cells (from Dr Timothy Adams, Commonwealth Scientific and Industrial Research Organisation) were adapted to grow in suspension in Freestyle 293 Expression medium (Life Technologies) supplemented with 200 mg l^−1^ G418 (Life Technologies) using a humidified shaker incubator (37 °C, 5% CO_2_, 130 r.p.m.). The adapted HEK293T culture was maintained in Erlenmeyer shaker flasks and scaled up in a 20-l WAVE bioreactor (GE Healthcare), seeded at an initial working volume of 5 l at a concentration of 0.8 × 10^6^ viable cells per ml. To reduce shear stress, Pluronic F86 (Life Technologies) was added to the culture at 0.2% w/v final concentration. The culture was scaled up to 20 l at a viable cell density of 3 × 10^6^ cells per ml, at which time transient transfection was initiated. The hTERT gene^54^ under a CMV promoter was cloned into plasmid pAPEX-3P^40^,^55^. The hTR gene under a U3 promoter^56^ and dyskerin (OriGene Technologies) under a CMV promoter were cloned into a single plasmid in vector pAPEX-3 (ref. 55). Plasmids were produced on the 200-mg scale by GenScript (USA) and used at an hTERT:hTR ratio of 1:19 by weight. Polyethylenimine (25 kDa linear, Polysciences) was prepared as an aqueous solution at 1 mg ml^−1^ at pH 7.0 and filter-sterilized (0.22 μm). Per litre of culture, a transfection mix was prepared by adding 1 mg DNA to 100 ml PBS pre-warmed to 37 °C, followed by addition of 4 ml polyethylenimine solution; the solution was mixed gently and incubated at room temperature (RT) for 15 min prior to addition to the 20-l culture, which was maintained at 37 °C with a rocking speed of 25 r.p.m. and rocking angle of 9°. At 2 days after transfection, the culture was fed with 5 g l^−1^ Lupin (Cell Biosciences), 2 mM Glutamax-1 (Life Technologies) and 5 g l^−1^ glucose (Sigma), and the temperature was reduced to 32 °C. The cells were harvested 4 days after transfection by centrifugation (1,500*g*, 10 min, 4 °C), snap-frozen on liquid nitrogen and stored at −80 °C. Telomerase high-expressing HEK293T cell pellets are available from Abbexa Ltd, Cambridge, UK.

### Purification of overexpressed 293T telomerase

Approximately 130 g 293T cell mass (∼4 × 10^10^ cells) was broken up to a paste in a 1-l plastic beaker. The cell paste was suspended in 800 ml ice-cold lysis buffer (10 mM HEPES-KOH (pH 8.0), 20 mM KCl, 2 mM MgCl_2_, 1% v/v Triton X-100, 1 mM dithiothreitol (DTT)). Once suspended, 8 ml of phenylmethylsulfonyl fluoride solution (100 mM in ethanol, made fresh) was added with stirring. The suspension was transferred in 50-ml portions to a 50-ml Dounce homogenizer with a tight pestal on ice and processed to ensure complete dissolution of cell paste, then pooled into a 1-l bottle equipped with magnetic stir bar. The lysate was stirred on ice for 1 h, then divided between four 250-ml bottles for the Beckman JA-14 rotor. The lysate was clarified with centrifugation at 14,000 r.p.m. (∼30,000*g*) at 2 °C for 30 min. The clear lysate (∼850 ml) was collected into a 1-l bottle equipped with magnetic stirrer. With stirring on ice, 22 ml of 2 M MgCl_2_ (∼50 mM final Mg^2+^) was added and the suspension was stirred for 1 h to selectively precipitate ribonucleoprotein complexes^57^. The suspension was divided between four 250-ml bottles for the Beckman JA-14 rotor, and the products were collected with centrifugation at 10,000 r.p.m. (∼15,000*g*) at 2 °C for 30 min. The supernatant was decanted to provide white pellets. Each pellet was suspended in 50 ml immunoprecipitation (IP) buffer (50 mM HEPES-KOH (pH 8.0), 500 mM KCl, 2 mM MgCl_2_, 1% v/v Triton X-100, 10% v/v glycerol and 1 mM DTT). To aid dissolution, the suspension was processed through a Dounce homogenizer with a tight pestal on ice until a slightly white, homogeneous solution was obtained. The material was pooled into a 500-ml bottle on ice, and the volume was made up to 300 ml with additional IP buffer. Polyclonal hTERT antibody (raised against hTERT amino acids 276–294 (ARPAEEATSLEGALSGTRH)^41^; available from Abbexa Ltd) (12 mg=40 μg ml^−1^ final concentration) was added and the solution shaken on ice for 30 min. To capture the antibody–telomerase complex, 12 ml of a 50% v/v slurry of Protein G/sepharose (GE Healthcare) was added (20 μl beads per ml final concentration) and the suspension was shaken on ice for 1.5 h. Working in a cold room, the immunoprecipitate was collected into a 50-mm diameter fritted glass column (Bio-Rad) with vacuum suction and then washed with 200 ml ice-cold IP buffer. The immunoprecipitate was suspended in 20 ml telomerase buffer (50 mM HEPES-KOH (pH 8.0), 300 mM KCl, 2 mM MgCl_2_, 0.1% v/v Triton X-100, 10% v/v glycerol and 1 mM DTT) containing 3 mg peptide ARPAEEATSLEGALSGTRH (20 molar equiv per antibody, available from Abbexa Ltd). The suspension was incubated at RT for 1 h with gentle shaking every 5 min, then the eluate was either collected (for use in assays requiring immunopurified telomerase) or transferred directly to a 25-mm diameter fritted glass column (Bio-Rad) containing 15 nmol gel-purified 5′-BIOTIN-CTAGACCTGTCATCA(TTAGGG)_3_-3′ oligonucleotide immobilized on 500 μl UltraLink Neutravidin Plus beads (Thermo-Fisher) (for the highly purified preparations in Figs 3d and 4b). The suspension was rotated at RT for 30 min and then at 4 °C for 1 h. For the work in this paper, it was necessary to elute telomerase in the presence of two different solution-phase DNA ‘traps', so that this DNA (traces of which remain in the purified telomerase preparation) was identical to the substrates being tested: 6 ml of suspension was collected in each of two 0.5-ml micro-spin columns (GE Healthcare), and washed with 3 ml cold telomerase buffer. After washing, columns were centrifuged for 10 s at 2,400 × *g* to remove residual buffer. Each bead sample was suspended in 500 μl telomerase buffer, followed by addition of 25 nmol of one of two different solution-phase DNAs dissolved in 100 μl telomerase buffer: (i) 7GGT (Table 1) or (ii) [7GGT]_4_ G-quadruplex. Activity-dependent elution was initiated by addition of 6 μl dTTP+dATP (10 mM each deoxyribonucleotide triphosphate, final concentration 0.1 mM). The suspensions were rotated at RT for 30 min and the product solutions collected with centrifugation at 2,400*g* for 10 s. Each product solution was layered on an 11-ml 10→40% glycerol gradient composed of (20 mM HEPES-KOH (pH 8.0), 300 mM KCl, 2 mM MgCl_2_, 0.1% w/v octyl β-D-glucopyranoside and 1 mM DTT) in Beckman Ultra-clear centrifuge tubes (14 × 89 mm) for the Beckman SW-41 rotor. Telomerase was sedimented with centrifugation at 35,000 r.p.m. (∼210,000*g* at *r*_max_) at 4 °C for 20 h. The tube was punctured at the bottom with a 30-gauge needle, and 0.5-ml fractions were collected by gravity. Fractions were assayed for telomerase concentration by dot-blot northern against hTR as described^40^, and equal amounts of enzyme used in each assay. Telomerase typically eluted in fractions 7–9; ∼3–5 pmol telomerase was obtained from each gradient. For extension assays using linear 7GGT or [7GGT]_4_ as substrates (Fig. 3d), the enzyme prepared in the presence of the equivalent ‘trap' DNA was used (with the [7GGT]_4_-trapped enzyme being used for the ‘No DNA' lanes). Enzyme trapped with linear 7GGT was dialysed into telomerase buffer containing 2.5 mM SrCl_2_, and equal amounts of this enzyme used for the extension of 22GGG and [22GGG]_2_ in Fig. 4b.

### SDS–PAGE and silver-staining analysis of purified telomerase

A 50-μl aliquot of purified telomerase, eluted in the presence of [7GGT]_4_ G-quadruplex DNA, was placed in a Pierce Slide-a-Lyzer mini-dialysis cup (MWCO 3,500) and dialysed at RT for 30 min against 200 ml of (20 mM HEPES-KOH (pH 8.0), 20 mM KCl, 2 mM MgCl_2_, 20% v/v glycerol, 0.1% w/v octyl β-D-glucopyranoside and 1 mM DTT). For SDS–PAGE, 30 μl of the dialysed solution was combined with 10 μl 10% w/v SDS, 5 μl 1 M DTT and 5 μl 4 × NuPAGE LDS loading buffer (Life Technologies). The sample was denatured at 80 °C for 10 min, cooled to RT and centrifuged at 16,000*g* for 1 min. A 20-μl aliquot was electrophoresed over a NuPAGE 4–12% bis-Tris gradient mini-gel at 100 V for 3 h. For the molecular weight marker, the Life Technologies Benchmark protein ladder was diluted 100-fold in 1 × NuPAGE LDS loading buffer; from this, 3 μl was diluted with 20 μl 1 × LDS buffer and loaded on the gel. Silver staining was performed with the Life Technologies SilverXpress kit.

### DHX36 western blotting

A 25-μl aliquot of immunopurified telomerase or four-step purified telomerase, eluted in the presence of [7GGT]_4_ G-quadruplex DNA, was combined with: 20 μl H_2_O, 25 μl 4 × NuPAGE LDS loading buffer, 10 μl 1 M DTT and 20 μl 10% w/v SDS. The samples were denatured at 80 °C for 10 min, cooled to RT and centrifuged at 16,000*g* for 1 min. A 30-μl aliquot of each was electrophoresed over a NuPAGE 4–12% bis-Tris gradient mini-gel at 100 V for 4 h. For the molecular weight marker, 2 μl of the Life Technologies MagicMark-XP was used. Proteins were transferred to polyvinylidene difluoride membranes at 30 V for 90 min. Blocking, washing, secondary anti-rabbit and chemiluminescent detection were performed using the Western Breeze reagents from Life Technologies. The primary anti-DHX36 antibody was polyclonal from rabbit, Abcam #70269, diluted 1:500 in primary antibody diluent; probing was performed at RT for 2 h. Figure 3c represents a 10-min exposure.

### Dyskerin western blotting

HEK293T cell pellets were resuspended in 4 × NuPAGE LDS loading buffer, 2% β-mercaptoethanol and 2% Benzonase nuclease (Merck Millipore) at 10,000 cells per μl. The samples were denatured at 68 °C for 10 min and centrifuged at 16,000*g* for 1 min. A 5-μl aliquot of each was electrophoresed over a NuPAGE 4–12% bis-Tris gradient mini-gel at 100 V for 2 h. For the molecular weight marker, 5 μl of the Bio-Rad Preision Plus prestained marker was used. Proteins were transferred to nitrocellulose membrane at 100 V for 60 min. Membranes were blocked using 5% skim milk, probed with primary antibodies diluted in 1% skim milk (mouse monoclonal anti-vinculin 1:5,000 (Sigma #V9131) or polyclonal rabbit anti-dyskerin (in-house) 1:1,000) for 1 h at RT and washed 5 × 5 min in TBST (0.24% Tris-Cl, 0.05% Tris, 0.8% NaCl and 0.1% Tween-20, pH 7.6). Membranes were then probed with horseradish peroxidase-conjugated secondary antibodies (goat anti-mouse or goat anti-rabbit; DAKO) diluted 1:5,000 in 1% milk. Detection was performed with Amersham ECL Prime western blotting detection reagent and a FujiFilm Las4000 luminescent image analyzer, with autoexposure.

### Telomerase activity assays

The following reaction was prepared to give 15 μl per sample: between 1 nM and 2 μM of the specified oligonucleotide, 50 mM Tris-HCl pH 8.5, 1 mM MgCl_2_, 5 mM DTT, 1 mM spermidine-HCl, 0.5 mM dTTP, 0.5 mM dATP, 4.6 μM non-radioactive dGTP and 0.33 μM [α-^32^P]dGTP at 20 mCi ml^−1^, 6,000 Ci mmol^−1^ (PerkinElmer Life Sciences), 10% glycerol and either 150 mM KCl for experiments with 7GGT or 2.5 mM SrCl_2_ for experiments with 22GGG. The reaction was initiated by adding 5 μl of purified human telomerase, and incubating at 30 °C for 1 h. The reaction was quenched by the addition of 80 μl of stop-buffer (50 mM Tris-HCl, pH 8.3, 20 mM EDTA and 0.2% SDS) and 1–2 × 10^3^ c.p.m. of a 5′-^32^P-labelled synthetic 100-mer DNA as an internal recovery standard. The solution was extracted with an equal volume of phenol/chloroform/isoamyl alcohol (25:24:1, v/v/v) and precipitated with ethanol in the presence of 2.5 M NH_4_OAc. The pellet was air-dried at RT for 15 min and dissolved in 5 μl TE buffer (10 mM Tris-HCl, pH 8.0 and 1 mM EDTA, pH 8.0), followed by addition of 5 μl formamide buffer (90% deionized formamide, 0.1% bromophenol blue and 0.1% xylene cyanol in 1 × TBE). The solution was heated at 90 °C for 5 min, and 3 μl was electrophoresed over a 10% polyacrylamide sequencing gel (0.2 mm thick × 40 cm length × 35 cm width, 32-well comb) run in 1 × TBE/8 M urea at 85 W. The gel was transferred to filter paper, dried for 30 min at 80 °C, exposed to a PhosphorImager screen, visualized on a Typhoon FLA9500 scanner (GE Healthcare Lifesciences) and analysed using ImageQuant software. The total intensities of extension products were normalized against the intensity of the ^32^P-labelled 100-mer recovery and loading control. Resulting values were expressed as a percentage of the reaction with maximal activity and plotted against substrate concentrations and fitted to the Michaelis–Menten equation to give *K*_m_ values.

For experiments where dTTP was titrated, the oligonucleotide concentration was kept at 1 μM and the concentration of dTTP was changed as outlined in the figure legend. All other reagents and procedures remained the same. Activity assays testing templated addition included 1–2 μM of the specified oligonucleotide and the following combinations and concentrations of labelled and unlabelled nucleotides; all other reaction conditions remained the same.

7GGT: T,A lanes: ^32^P-dTTP (25 μM, 80 Ci mmol^−1^) and dATP (0.5 mM).

T,A,ddG lanes: ^32^P-dTTP (25 μM, 80 Ci mmol^−1^), dATP (0.5 mM) and ddGTP (0.5 mM).

T,A,G lanes: ^32^P-dTTP (25 μM, 80 Ci mmol^−1^), dATP (0.5 mM) and dGTP (0.5 mM) or dTTP (0.5 mM), dATP (0.5 mM) and ^32^P-dGTP (5 μM, 400 Ci mmol^−1^).

22GGG:T,A lanes: ^32^P-dTTP (3 μM, 150 Ci mmol^−1^) and ddATP (25 μM).

T,A,ddG lanes: ^32^P-dTTP (3 μM, 160 Ci mmol^−1^), dATP (0.5 mM) and ddGTP (25 μM).

T,A,G lanes: dTTP (0.5 mM), dATP (0.5 mM) and ^32^P-dGTP (5 μM, 400 Ci mmol^−1^).

Experiments in which reaction products were subjected to native gel electrophoresis contained 2 μM DNA, 0.7 μM non-radioactive dTTP and 0.33 μM [α-^32^P]dTTP at 10 mCi ml^−1^, 3,000 Ci mmol^−1^, with all other reaction conditions remaining the same. The reaction was quenched by the addition of 20 μl of stop-buffer (see above) and 8 × 10^3^ c.p.m. of a 5′-^32^P-labelled synthetic 100-mer DNA as an internal recovery standard. The product was purified of unincorporated deoxyribonucleotide triphosphates using two rounds of mini Quick Spin Oligo column purification (Roche). Prior to use, Quick Spin Oligo columns were equilibrated by centrifugation twice at 1,000g for 10 min with 400 μl Sr^2+^ hTel buffer (see above). The purified product was added to 6 × native gel loading buffer (see above) and electrophoresed on a non-denaturing 16% polyacrylamide gel containing 2.5 mM SrCl_2_ for ∼48 h at 40 V at 18 °C. Both buffer and gel contained the same constituents as the DNA-folding buffer. The gel was transferred to filter paper, dried for 30 min at 80 °C, exposed to a PhosphorImager screen, visualized on a Typhoon FLA9500 scanner and analysed using ImageQuant software.

### Expression and purification of G-quadruplex antibody (BG4)

BG4-encoding plasmid (from the laboratory of Professor Shankar Balasubramanian, University of Cambridge, UK) was transformed into BL21(DE3) competent cells (Stratagene), which were cultured in TY media (1.6% tryptone peptone, 1% yeast extract and 0.5% NaCl) and 50 μg ml^−1^ kanamycin. BG4 antibody expression was induced with 0.5 mM isopropyl β-D-1-thiogalactopyranoside for 3 h at 37 °C. The cells were lysed in TES buffer (50 mM Tris-Cl pH 8.0, 1 mM EDTA and 20% sucrose) on ice for 10 min, diluted twofold in water, and left on ice for a further 10 min prior to centrifugation at 10,000*g* at 4 °C for 30 min. The supernatant was filtered (0.2 μm) and purified on a HIS-Select Nickel Affinity column (Sigma). The column was washed in 10 mM imidazole pH 8.0 in PBS, and BG4 antibody eluted in 250 mM imidazole pH 8.0 in PBS. BG4 antibody was concentrated and buffer exchanged into PBS in an Amicon Ultra-15 Centrifugal Filter Unit (Millipore). The concentration of BG4 was determined using Thermo Scientific Pierce BCA Protein Assay kit, and the antibody was stored at −20 °C.

### Cell synchronization and short interfering RNA (siRNA) transfection

HEK293T mid-S-phase cell synchronization was performed using cells released from a thymidine/aphidicolin block as previously described^49^. Dyskerin knockdown was performed by reverse transfecting 120 pmol of Invitrogen custom-designed Stealth siRNA targeting either the dyskerin 3′ untranslated region (siDKC#1, 5′-AAGGCCACUUGAAGCUGGAGGAGAA-3′) or the coding region (siDKC#2, 5′-GGCCAAGATTATGCTTCCAGGTGTT-3′). Cells were transfected using Life Technologies Lipofectamine RNAiMAX Transfection Reagent. Qiagen All Stars negative-control siRNA was used as a siRNA control.

### Immunofluorescence and telomere/telomerase FISH

Immunofluorescence and FISH for telomeres and telomerase RNA (hTR) were performed as described^49^, with the following modifications. Cytoplasm was removed by incubating slides in cytoplasmic extraction buffer (20 mM HEPES-KOH, pH 7.9, 20 mM NaCl, 5 mM MgCl_2_, 300 mM sucrose and 0.5% (v/v) NP-40) for 10 min with no shaking. Slides were washed once with PBS+0.1% Tween-20, once with PBS and then fixed in 2% paraformaldehyde in PBS for 20 min. Cells were washed with PBS then incubated in 0.1% Triton X-100 in PBS for 10 min, followed by rinsing twice in PBS. Slides were ethanol dehydrated with 70% (2 min), 90% (2 min) and 100% ethanol (2 min) followed by air drying prior to overlaying with 30 μl of FISH buffer containing 5 ng each of five Alexa Fluor 488-labelled anti-hTR oligonucleotides and 5 ng of Texas Red-labelled telomere probe^49^. Slides were heated to 80 °C for 3 min and incubated in a humidified chamber overnight at 37 °C and washed^49^. Slides were refixed in 2% paraformaldehyde in PBS, washed as before and overlaid with phosphate-buffered gelatin^49^ for 1 h. Slides were incubated with BG4 primary antibody (600 nM) for 1 h at 37 °C, washed four times for 5 min with PBS+0.01% Tween-20 and then overlaid with secondary antibody (Rabbit anti-DYKDDDDK Tag Antibody, Cell Signalling; 1:800 dilution) for 1 h at 37 °C. Cells were washed as before and a fluorescently labelled tertiary antibody (Alexa Fluor 647 Donkey Anti-Rabbit IgG (H+L) Antibody, Life Technologies; 1:1,000 dilution) was overlaid at 37 °C for 30 min, washed as before and counterstained with 4′,6-diamidino-2-phenylindole. Staining was visualized at RT on a Zeiss Axio Imager M1 microscope, with a Plan-Apochromat × 63 oil objective (numerical aperture, 1.4), and an AxioCam MR digital camera (Carl Zeiss) with consistent exposure times between experiments. For presentation purposes, pixel intensity histograms were adjusted in Axiovision (Carl Zeiss), equally across all figure panels, and images were cropped in Adobe Photoshop.

## Additional information

**How to cite this article:** Moye, A. L. *et al.* Telomeric G-quadruplexes are a substrate and site of localization for human telomerase. *Nat. Commun.* 6:7643 doi: 10.1038/ncomms8643 (2015).

## Supplementary Material

Supplementary Information Supplementary Figures 1-7, Supplementary Table 1 and Supplementary References

## Figures and Tables

**Table 1:** Oligonucleotides used and G-quadruplex structures characterized in this study.

**Nomenclature**	**Sequence (5′–3′)**	**Length (nt)**	**G4 nomenclature**	**Annealing cation**	**Strand orientation**	**Strand stoichiometry**	***T***_**½**_ **(°C)**
7GGT	TTAGGGT	7	[7GGT]_4_	150 mM K^+^	Parallel	Tetramer	68
22GGG	AGGG(TTAGGG)_3_	22	[22GGG]_2_	2.5 mM Sr^2+^	Parallel	Dimer	>90
22GGG	AGGG(TTAGGG)_3_	22	[22GGG]_1_	2.5 mM Sr^2+^	Antiparallel	Intramolecular	68
9C	CCCTAACCC	9					
T_15_	TTTTTTTTTTTTTTT	15					
T_25_	TTTTTTTTTTTTTTTTTTTTTTTTT	25					
