# Supplementary material for: Telomeric G-quadruplexes are a substrate and site of localization for human telomerase
Source: Nat Commun. 2015 Jul 9;6:7643. doi: 10.1038/ncomms8643 (PMC4510649; doi:10.1038/ncomms8643)
Supplement: Supplementary Information — Supplementary Figures 1-7, Supplementary Table 1 and Supplementary References [file ncomms8643-s1.pdf]

Supplementary Figure 1

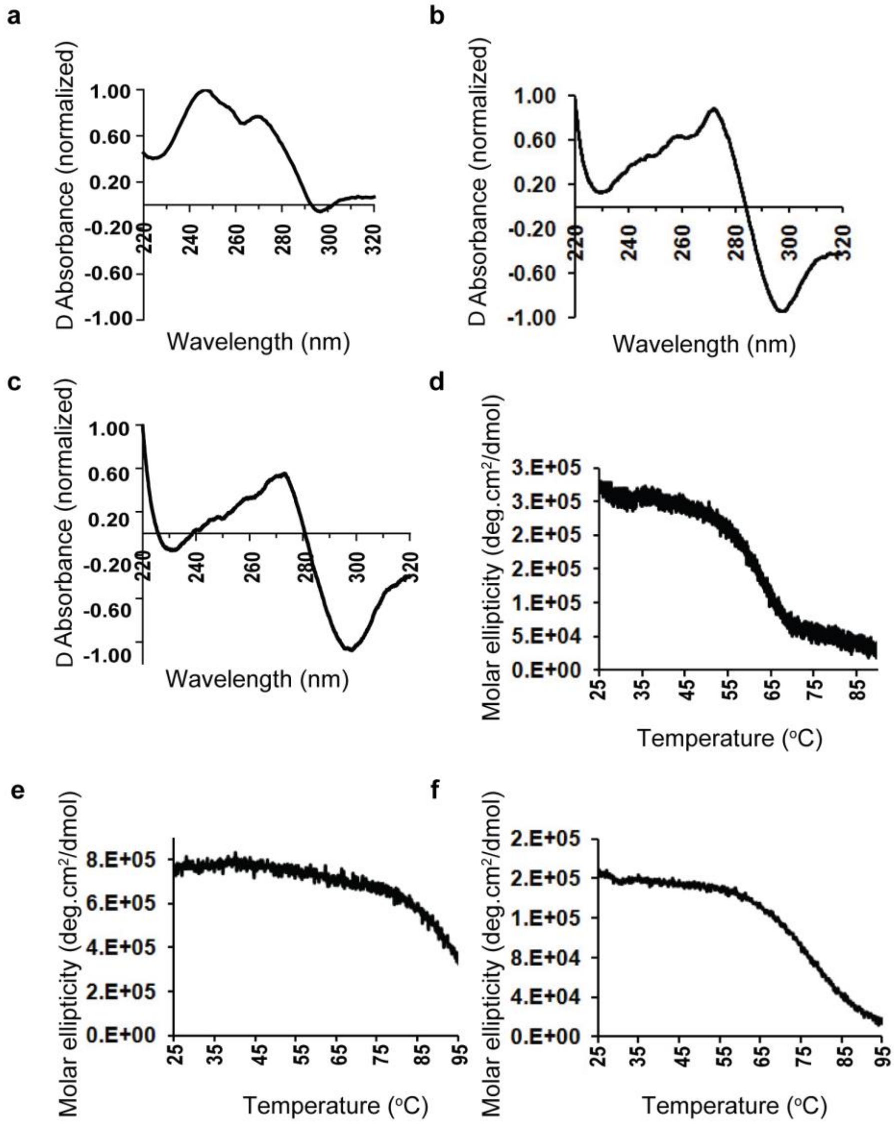

Supplementary Figure 1: Difference spectra and melting profiles of G-quadruplexes

(**a**) Normalized UV isothermal difference spectrum (IDS) of $[7GGT]_4$ in 150 mM K^+ hTel buffer at 20 µM DNA concentration. A thermal difference spectrum (TDS) requires obtaining one UV spectrum at an elevated temperature so that the G-quadruplex is unfolded, and one at a lower (generally 25˚C) temperature[1]. However, because of the slow rate of formation of tetrameric G-quadruplexes such as $[7GGT]_4$[2], we were able to obtain the folded and unfolded spectra at room temperature. In these instances the resulting difference spectrum is called IDS.
(**b**) and (**c**) Normalized UV thermal difference spectrum (TDS) of the gel purified upper band of folded 22GGG (**b**), or lower band of folded 22GGG (**c**) in 2.5 mM Sr^{2+} hTel buffer at 20 µM DNA concentration.
(**d**) Melting profile of $[7GGT]_4$ in 150 mM K^+ hTel buffer at 20 µM DNA concentration recorded by CD at 260 nm and heated at 1˚C min^{-1}.
(**e**) Melting profile of gel-purified upper band of folded 22GGG in 2.5 mm Sr^{2+} hTel buffer at 20 µM DNA concentration, recorded by CD at 260 nm and heated at 1˚C min^{-1}.
(**f**) Melting profile of gel-purified lower band of folded 22GGG in 2.5 mM Sr^{2+} hTel buffer at 20 µM DNA concentration, recorded by CD at 260 nm and heated at 1˚C min^{-1}.

Supplementary Figure 2

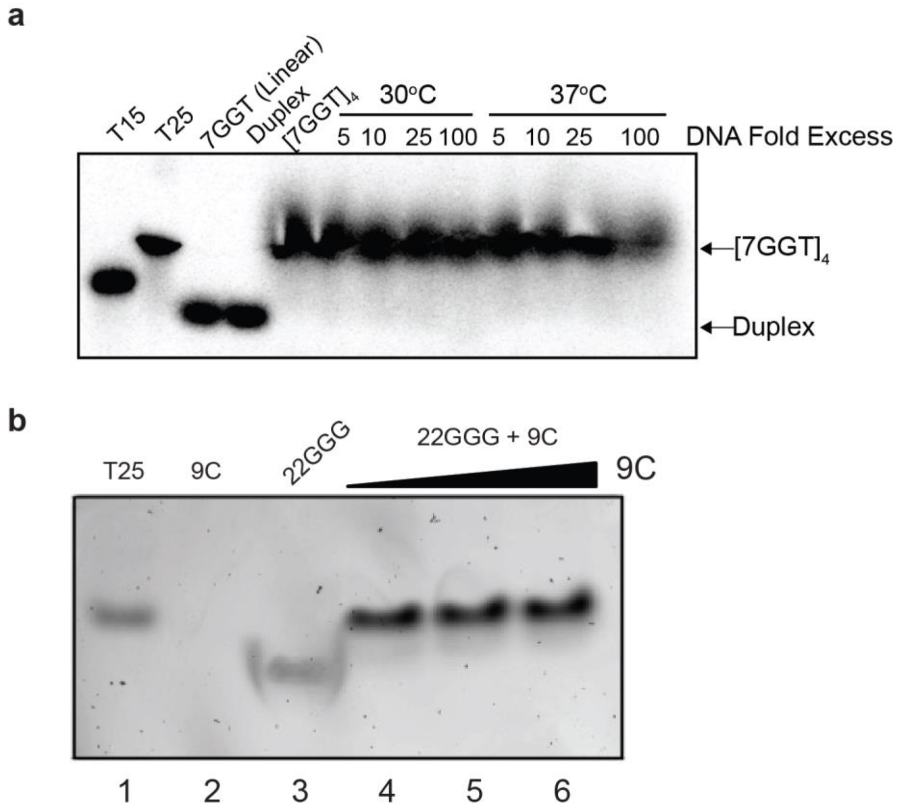

Supplementary Figure 2: C-strand binding controls

(a) G4 unfolding is rate limiting for DNA-9C duplex formation. $[7GGT]_4$ complementary strand trap assay: 5´ end-labeled $[7GGT]_4$ (11 μM) in 150 mM K+, incubated with 5 to 100-fold excess complementary strand 9C at 30°C for 4 h and electrophoresed on a 12% nondenaturing polyacrylamide gel. Markers include the unstructured MW markers T_{15} and T_{25}, denatured 7GGT, pre-annealed WC duplex and $[7GGT]_4$ G-quadruplex.

(b) SYBR® Gold stained native gel demonstrating hybridization of the C-strand oligonucleotide 9C to 22GGG. Lane 1: unstructured T_{25} MW marker. Lane 2: 9C; note that this oligonucleotide either does not take up SYBR® Gold or runs off the gel. Lane 3: denatured 22GGG. Lanes 4-6: 250 ng 22GGG pre-annealed with 2-, 5- and 10-fold excess 9C.

Supplementary Figure 3

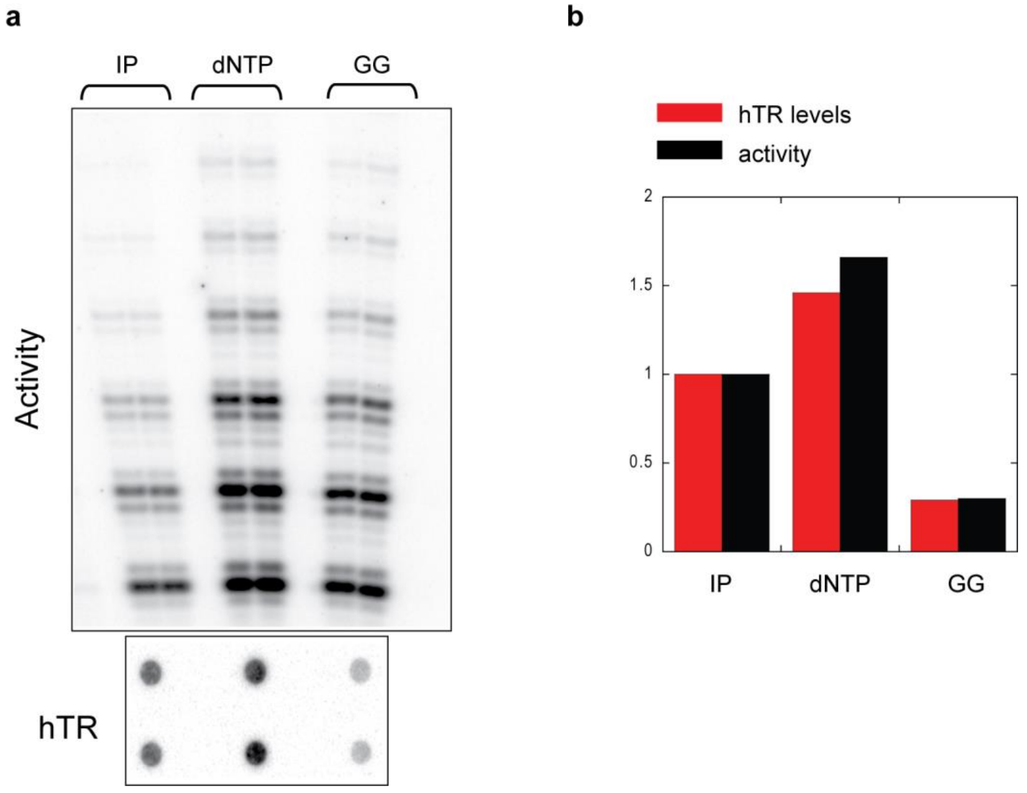

Supplementary Figure 3: Specific activity of telomerase at various stages of purification

(**a**) Upper panel: Telomerase activity assay using an 18 nt DNA substrate $(TTAGGG)_3$, in duplicate. Samples are equal volumes of 293T lysate after immunopurification with an hTERT antibody (IP), after activity-dependent elution from immobilized substrate (dNTP) and after a glycerol gradient (GG). The same volumes of each fraction were loaded in duplicate on a dot blot which was probed for hTR (bottom panel).

(**b**) Quantitation of data in (**a**). Activity and hTR levels were normalized to the levels in the IP sample. Note that activity relative to hTR levels, i.e. specific activity, remains constant over the purification.

Supplementary Figure 4

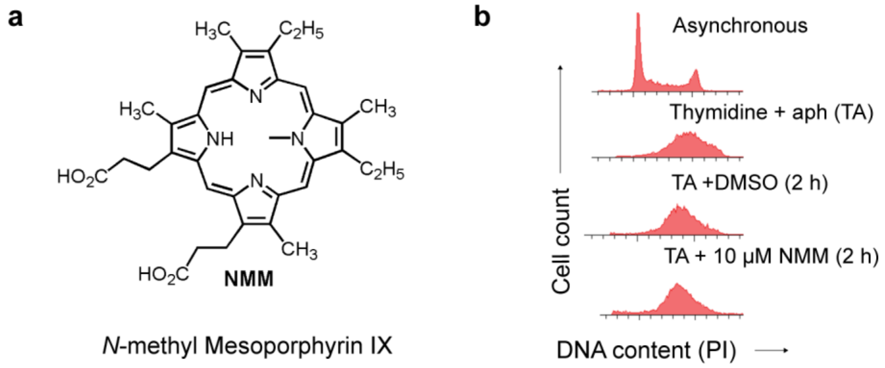

Supplementary Figure 4: *N*-methyl Mesoporphyrin IX treatment of 293T cells does not affect progression into mid-S phase of the cell cycle

(**a**) Chemical structure of the parallel G-quadruplex specific ligand *N*-methyl mesoporphyrin IX (NMM).

(**b**) Flow cytometry analysis demonstrates that vehicle only (DMSO) and NMM treatment of HEK293T cells did not effect the cells' ability to enter S phase. Cells were sorted on the basis of DNA content as determined by staining with propidium iodide (PI). Cell populations were asynchronous, or synchronized at the G1/S border with thymidine/aphidicolin treatment (TA), then released and allowed to progress into S phase for 3 h.

Supplementary Figure 5

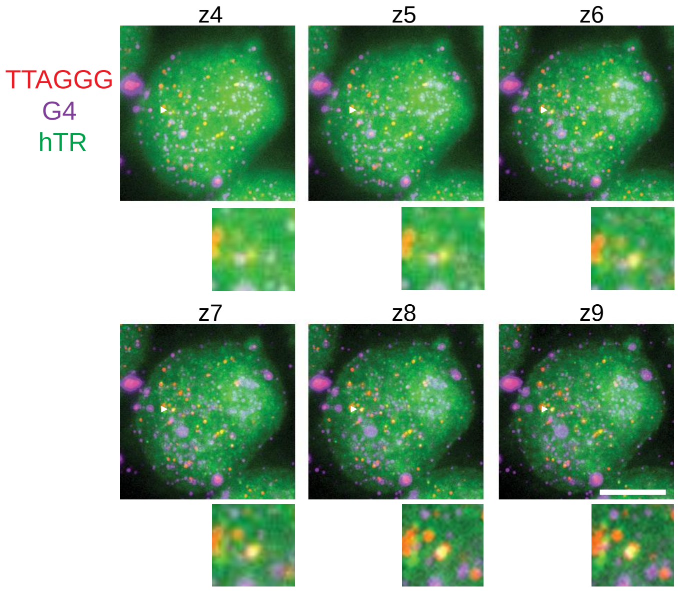

Supplementary Figure 5: Deconvolution microscopy of hTR-telomere-G-quadruplex trilocalizations

Representative HEK-293T nuclei z-stacks showing that G-quadruplex (purple), telomere (red) and telomerase (hTR, green) trilocalization (yellow) is not due to random overlap. Six of the ten captured planes through the nucleus (z4 – z9) show that all three colors localize to the same plane of the cell.

Supplementary Figure 6

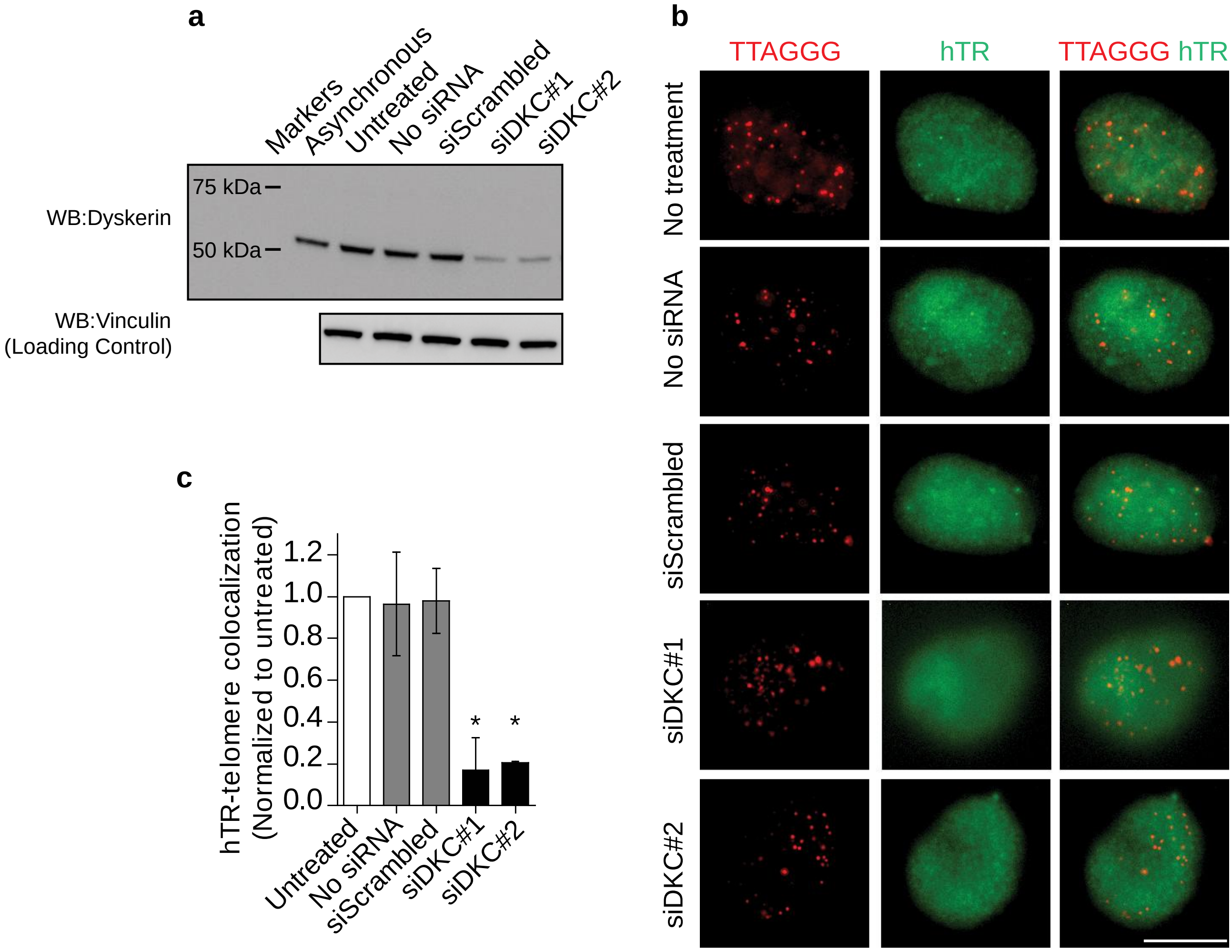

Supplementary Figure 6: Reduction of hTR FISH foci by siRNA knockdown of dyskerin

(a) Western blot showing dyskerin knockdown using siDKC#1 (directed against the 3′ UTR) and siDKC#2 (directed against the coding region), in mid-S phase HEK-293T cells. Vinculin: loading and recovery control. No siRNA: transfection reagent only control (Lipofectamine® RNAiMAX). siScrambled: Qiagen All Stars negative control siRNA.

(b) Representative mid-S phase HEK293T nuclei demonstrating reduction in hTR foci (green) following siRNA-mediated dyskerin knockdown (n=2). No siRNA: transfection reagent only control (Lipofectamine® RNAiMAX), siScrambled: Qiagen All Stars negative control siRNA. Scale bar = 10 µM.

(c) Quantitation of hTR (telomerase, green) localization at telomeres (red) following the treatments in (**a**) and (**b**). Colocalization events were normalized to untreated samples. Error bars represent SD for n=2 experiments. Statistical significance was determined using an unpaired student t-test, relative to siScrambled. **P*<0.05

Supplementary Figure 7

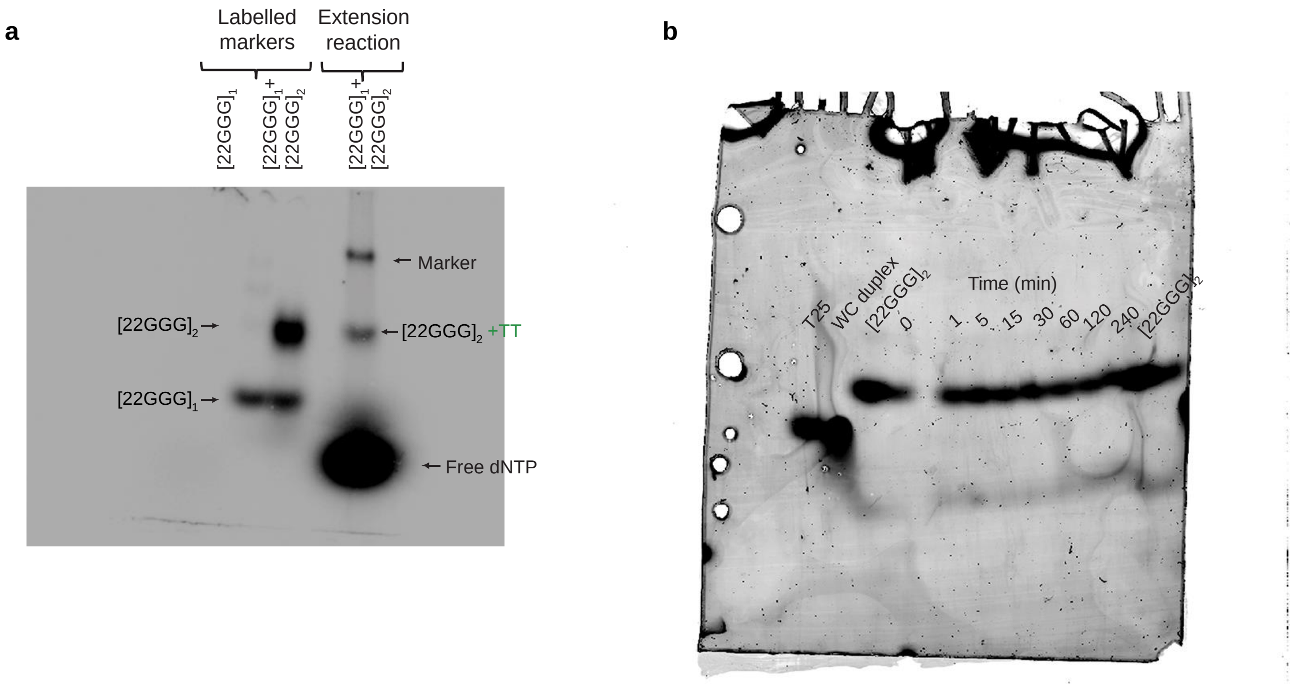

Supplementary Figure 7: Uncropped gels and blots from manuscript

(a) Uncropped native gel electrophoresis image presented in Figure 5c.

(b) Uncropped $[22GGG]_2$ complementary C-strand experiment presented in Figure 2d.

Supplementary Table 1: Assignment of ESI-MS ions to species of oligonucleotide 22GGG (MW = 6966.5 Da) based on calculated *m/z* values.

**Expected ions***	**MW of ion (Da)**	**Calculated *m/z***	**Observed *m/z***
$[M-5H]^{5-}$	6961.5	1392.3	1392.6
$[M-4H]^{4-}$	6962.5	1740.6	1741.0
$[M+Sr^{2+}-7H]^{5-}$	7047.1	1409.4	1409.8
$[M+Sr^{2+}-6H]^{4-}$	7048.1	1762.0	1762.5
$[2M+3Sr^{2+}-13H]^{7-}$	14182.8	2026.1	2026.6
$[2M+3Sr^{2+}-14H]^{8-}$	14181.8	1772.7	1773.2

*M is the mass of neutral, unimolecular 22GGG.

Supplementary References

1. Mergny,J.L., Li,J., Lacroix,L., Amrane,S., & Chaires,J.B. Thermal difference spectra: a specific signature for nucleic acid structures. *Nucleic Acids Res.* **33**, e138 (2005).
2. Mergny,J.L., De Cian,A., Ghelab,A., Sacca,B., & Lacroix,L. Kinetics of tetramolecular quadruplexes. *Nucleic Acids Res.* **33**, 81-94 (2005).
